# The Art and Science of Kidney Transplant Offer Evaluation

**DOI:** 10.7759/cureus.43223

**Published:** 2023-08-09

**Authors:** Nikhil A Reddy, Ashraf I Reyad, Sridhar R Allam

**Affiliations:** 1 North Texas Division, HCA Healthcare Research Institute, Fort Worth, USA; 2 Transplant Surgery, Medical City Fort Worth Transplant Institute, Fort Worth, USA; 3 Transplant Nephrology, PPG Health, Fort Worth, USA

**Keywords:** kidney allocation system, kidney donation, kidney donor profile index, organ utilization, kidney transplant

## Abstract

Currently, there are more than 100,000 patients on the transplant waitlist in the United States. There exists a significant gap between the supply and demand for kidney transplants. Despite this, about a quarter of kidneys recovered from deceased donors are not being utilized. There is a significant variation in kidney acceptance criteria by transplant centers. The current kidney allocation system allows transplant centers to place kidneys into appropriate recipients who may not be at the top of the list to increase organ utilization. A recent study questioned this practice of “list diving.” In this editorial, we seek to support “list diving” through a discussion of the various factors a transplant center could take into consideration while evaluating organ offers.

## Editorial

A recent study by King et al. [1] reported that transplant centers frequently skip candidates with the highest priority on the transplant waitlist. This finding could send a message of violation of the transplant waitlist concept, undermining trust in the system. Kidney Donor Profile Index (KDPI) is used as a measure of organ quality and Estimated Post Transplant Survival (EPTS) is used as a measure of recipient longevity in the study. However, these indices are only moderately accurate with c-statistics of 0.60 for KDPI [2] and 0.69 for EPTS [3].

When evaluating organ offers, transplant clinicians also consider several other factors that are not included in these indices. Kidneys with marginal biopsy findings offered to recipients with the highest priority may not be in the recipients’ best interest, given they are likely to receive better offers in the near future. Technical considerations exist: kidneys with multiple vessels are not optimal for recipients with extensive vascular calcifications, and kidneys requiring repair of anatomic damage or vascular reconstruction would render some recipients ineligible candidates. Pediatric donor kidneys may not be suitable for adult recipients at risk of hyperfiltration injury such as those with a high body mass index or a history of focal segmental glomerulosclerosis, or those at an increased risk of thrombosis from a known hypercoagulable state. Smaller kidneys offered to larger recipients may lead to inadequate nephron dosing and suboptimal long-term kidney function. Kidneys at risk of delayed graft function (DGF) should likely not be offered to recipients who are not predicted to tolerate DGF. Pre-emptive candidates whose current glomerular filtration rate (GFR) and recent GFR slope indicate no imminent need for dialysis can wait longer for a better offer. The travel time that candidates can take to come to the transplant center is also considered, especially for kidneys with long cold ischemia time. Lastly, some organ offers may be turned down by informed recipients if they do not see such offers as the best fit for themselves.

Transplant teams spend a significant amount of time evaluating organ offers and candidates on the match run to make the best possible decisions with the interest of patients always at the forefront, even those who may be bypassed. Under current practice, our center, while maintaining the highest tier ratings for survival on the waitlist, transplant rate, and one-year post-transplant graft survival, has been able to achieve a high offer acceptance ratio of 1.38 vs. 0.69 for our Organ Procurement Organization (OPO) [4]. For hard-to-place kidneys, the acceptance ratio is 1.08 for our center vs. 0.44 for our OPO. Accordingly, OPOs are obligated to offer these kidneys to aggressive centers in an expedited manner to abate long cold ischemia time that could further jeopardize the acceptance of these organs.

While there is a need for improving organ allocation efficiency, there are several factors that impact the organ acceptance decision-making process. We are not certain if all of these can be included in an objective index to help allocate kidneys more efficiently. We believe, for the most part, transplant programs are doing their best to match the right organ to the right recipient to reduce organ discard. Increased regulatory pressure to strictly follow the currently imperfect allocation match run process, and not apply clinical discretion and the very art of medicine, could have negative consequences on organ utilization. This could ultimately hurt the very patients we are all trying to help.

Novel solutions, however, appear promising in improving current inefficiencies. Technological innovations such as artificial intelligence (AI) and machine learning (ML) are rapidly evolving in medicine [5]. In the current age of transplantation, donors and recipients are increasingly complex. Additionally, there is wide variation in transplant center practices. Given the large datasets within the field of transplantation, AI and ML may hold great promise in improving the efficiency of both organ allocation and acceptance.
